# Loneliness in the Norwegian adolescent population: prevalence trends and relations to mental and self-rated health

**DOI:** 10.1186/s12888-023-05404-5

**Published:** 2023-11-30

**Authors:** Nayan Parlikar, Kirsti Kvaløy, Linn Beate Strand, Geir Arild Espnes, Unni Karin Moksnes

**Affiliations:** 1 Department of Public Health and Nursing, Faculty of Medicine and Health Sciences, Norwegian University of Science and Technology, Trondheim, Norway; 2 HUNT Research Centre, Department of Public Health and Nursing, Faculty of Medicine and Health Sciences, Norwegian University of Science and Technology, Levanger, Norway; 3 Levanger Hospital, Nord-Trøndelag Hospital Trust, Levanger, Norway; 4 Centre for Sami Health Research, Department of Community Medicine, UiT - The Arctic University of Norway, Tromsø, Norway

**Keywords:** Adolescence, Loneliness, Prevalence, Trends, Risk factors to loneliness, Mental distress, Self-rated health

## Abstract

**Background:**

Loneliness has become a significant public health problem and should be addressed with more research over a broader period. This study investigates the variations in the prevalence of loneliness among a nationally representative study population of Norwegian adolescents over the last three decades and whether age, gender, self-rated health, and mental distress are associated with these changes.

**Methods:**

Adolescents aged 13–19 years completed the structured and validated questionnaires from the three waves of the Young-HUNT Study: 1995–1997, 2006–2008, and 2017–2019. Loneliness was measured with one item asking, ‘Are you lonely?’. Hopkins Symptom Checklist-5 was used to measure mental distress (cut-off ≥ 2). Self-rated health was assessed by a single question ‘How is your health at the moment?’ Measures were provided by self-report. Descriptive analyses were stratified by age, gender, self-rated health, and mental distress. Linear-by-Linear association test across survey years was performed to test time trends of loneliness. Logistic regression was used to analyze the cross-sectional associations of self-rated health and mental distress with loneliness, adjusting for sociodemographic factors in all three waves of Young-HUNT.

**Results:**

Loneliness prevalence doubled from 5.9% in 1995/97 to 10.2% in 2017/19 in the total population sample. The highest loneliness prevalence and an increase from 8.9% in 1995/97 to 16.7% in 2017/19 was observed in girls of 16–19 years. Among mentally distressed adolescents, loneliness increased from 22.3% in 1995/97 to 32.8% in 2006/08 and lowered to 27% in 2017/19. Increasing loneliness prevalence was seen in those with poor self-rated health, i.e., 14.6% in 1995-97 and 26.6% in 2017-19. Mental distress and poor self-rated health were associated with higher odds of loneliness in each wave (p < 0.001).

**Conclusion:**

The results highlight the increasing burden of loneliness in the Norwegian adolescent population, especially girls. Those with mental distress and poor self-rated health have a higher risk of experiencing loneliness. Thus, health-promoting upbringing environments for children and adolescents that support mutual affinity, social support, integration, and belongingness in adolescents’ daily arenas are essential.

**Supplementary Information:**

The online version contains supplementary material available at 10.1186/s12888-023-05404-5.

## Background

Loneliness is considered a public health concern worldwide [1], and even a global epidemic [2]. Loneliness is a multidimensional experience defined as the emotionally unpleasant state arising from the perception of a lack of desired interpersonal relationships quantitatively or qualitatively [3, 4]. Loneliness is also defined as an individual experience of unpleasant or inadmissible lack of quality in certain relationships [5]. Research suggests that loneliness can be divided into two dimensions: social and emotional [6–8]. Social loneliness refers to the experienced absence of a social network, whereas emotional loneliness refers to the experienced lack of intimate and emotional attachment. Considerable evidence suggests that adolescence seems to be a particularly vulnerable period where one may experience loneliness more frequently and evidently than in later life periods due to being in a state of biological, psychological, social, and cognitive development and transition [9–11]. The young gradually seek independence from family and instead attempt to develop social and emotional bonds with their peers [11]. A lack of attaining intimate relationships with peers in this period of life can increase the risk of experiencing loneliness [12]. Because the evolution from childhood to adulthood is often associated with social pressure and insecurity, the feeling and risk of loneliness are crucial to focus on [13–15] and have therefore long been considered an important research topic and public health issue.

The increased experience of loneliness appears to be a worldwide phenomenon. Loneliness increased significantly between 2010 and 2017 among U.S. adolescents, particularly among girls. The mean loneliness score among girls increased from 2.23 in 2010 to 2.54 in 2017 and from 2.23 in 2010 to 2.44 in 2017 among boys [16]. Twenge et al. show an increase in adolescent loneliness in English-speaking countries after 2012. In a sample of one million adolescents, school loneliness increased between 2012 and 2018 in 36 out of 37 countries worldwide [17]. In a recent meta-analysis of adolescents 12–17 years in 2019 and 2020, the pooled prevalence of adolescent loneliness ranged from 9.2% in South-East Asia to 14.4% in the Eastern Mediterranean region [18]. A descriptive study [19] from 2017/2018 conducted in the four Nordic countries, Iceland, Sweden, Denmark, and Finland, found that 14% of adolescents in the Nordic countries reported feeling lonely frequently. The prevalence of loneliness was shown to be highest in Finland (19.2%) and Iceland (17.1%) and lowest in Denmark (7.7%). Girls reported approximately twice the rates of loneliness compared to boys in all four Nordic countries. A Finish study among 11-15-year-olds observed an escalating tendency of loneliness between 2006 and 2018 [20]. The prevalence of frequent loneliness increased from 15% in 2006 to 19% in 2018 among girls and from 7% in 2006 to 10% in 2018 among boys. Frequent loneliness was particularly prevalent, especially among 15-year-old girls, of whom 25% reported experiencing frequent loneliness in 2018. The National Norwegian survey study, Ungdata, has revealed that the prevalence of loneliness among adolescents increased from 8% in 2010 to 11% in 2021, where approximately twice as many girls as boys report loneliness [21, 22]. However, very few population-based time-trend studies over extensive time durations on adolescent loneliness dating back to the 1990s have been published in Norway. There is, therefore, a need to provide a more comprehensive picture of loneliness and how it has changed over a more extended period in the Norwegian adolescent population.

Loneliness has been linked to risk factors such as low socioeconomic status [23], small social networks [24], and parental divorce [25]. Most studies show that female sex is a significant predictor of loneliness [20, 26]. Nevertheless, new investigations are needed to understand the role of gender and age in adolescents’ experience of loneliness as the results of studies focusing on the gender associations of loneliness are inconsistent. Body image dissatisfaction, low self-esteem, social vulnerability, internalization of problems among girls [20, 27] and downplay of emotions, lack of friendships, and reluctance to feelings among boys are some of the factors associated with different gender patterns of loneliness [28, 29]. Regarding the age-related associations, previous studies have found evidence for a U-shaped curve across the life span, with peak loneliness scores during adolescence and old age [30–33]. For example, a recent study from Finland examined loneliness among 11-, 13-, and 15-year-old adolescents at four-year intervals (2006, 2010, 2014, 2018). The results showed that the proportion experiencing frequent loneliness increased from 11 to 15% over the 12-year study period, particularly among 15-year-olds [20]. However, there is a need for further knowledge concerning loneliness variance related to age groups.

The knowledge of the prevalence of loneliness among young people with symptoms of depression and poor health status is inadequate. Adolescence is a period characterized by higher severity of reported mental health problems than other phases of life [34, 35]. During the past two decades, there has been a substantial increase in child and adolescent mental health problems [16, 36–40] and qualitative studies describe a two-way association between loneliness and depression [35]. This means that lonely young people are more prone to be depressed, but also that their depression stimulates their loneliness and its negative impact [41]. However, there is a limited understanding of how these factors are associated.

Similarly, bidirectional association is observed between loneliness and subjective health; loneliness may adversely impact health through a variety of behavioural, mental, and physiological ways [42, 43], while poor or worsening health may hinder social interaction, which in turn intensifies loneliness impose hindrances [44]. Most of these studies have been performed in adults, and insufficient attention has been given to investigating prevalence time trends, gender and age differences, and associations between mental and subjective health and loneliness in the adolescent population. Such research is needed to attain a comprehensive understanding of the phenomenon and stimulate public health policy development and implementation of public health strategies.

This study, therefore, aimed (1) to observe the overall prevalence trend of loneliness in Norwegian adolescents from 1995 to 2019 and how these trends vary over time in relation to gender, age, mental distress, and self-rated health; and (2) to explore the associations of mental distress and self-rated health with adolescent loneliness. Based on the literature reviewed, we hypothesized that (1) loneliness prevalence increased over the measured time period (2) trends over time would change significantly according to gender, age mental health, and self-rated health and (3) adolescents with mental distress and poor self-rated health would be at a higher risk of experiencing loneliness.

## Materials and methods

### Study population

**Fig. 1:**
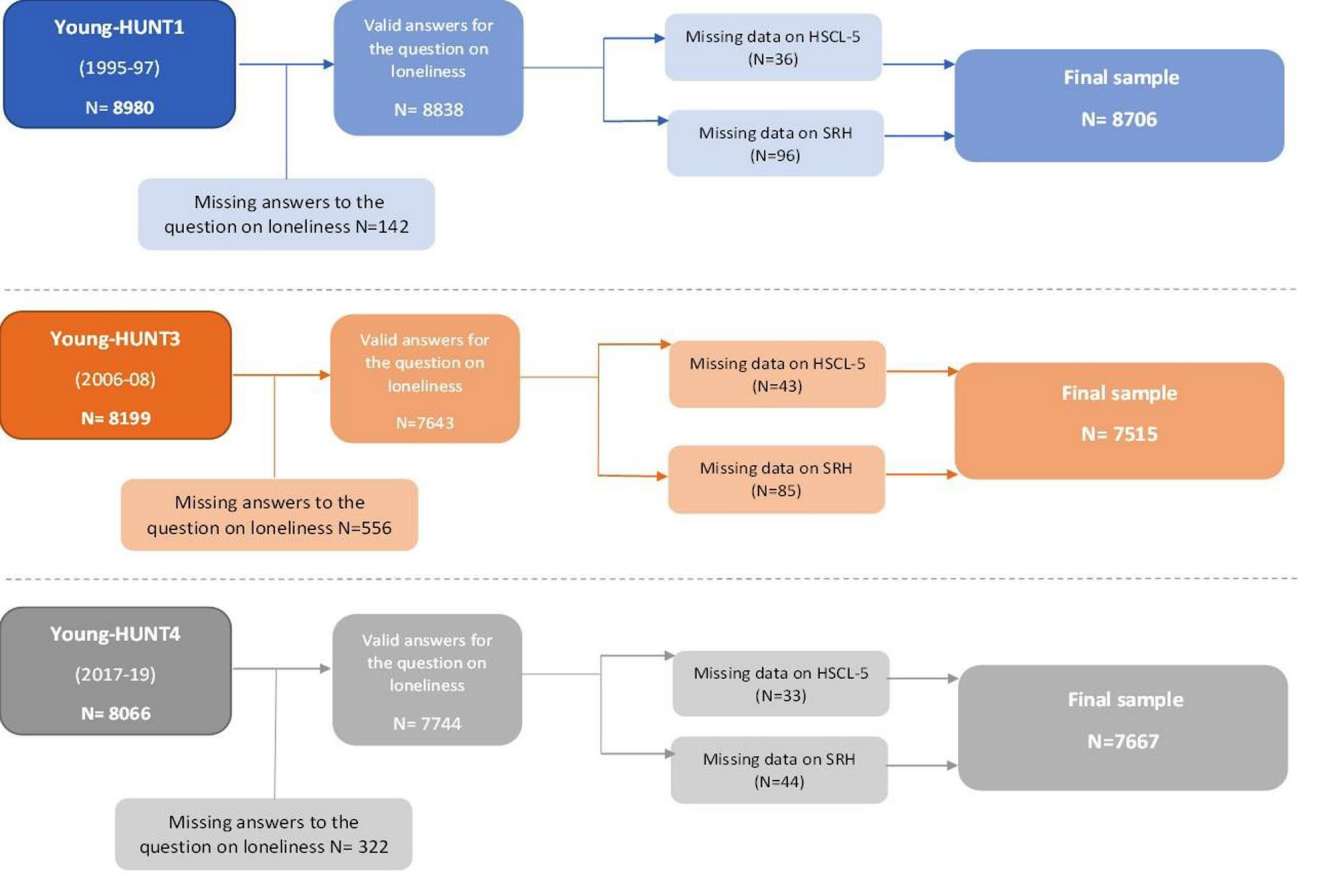
Flow chart of the number of study participants. 5-item Hopkins Symptom Checklist (HSCL-5); Self-rated health (SRH)

The Young-HUNT Study [45] is the adolescent part of the population-based Health Study of Trøndelag (The HUNT Study), which includes adolescents aged 13–19 years residing in the northern part of Trøndelag County in Norway. The data included are from three different waves in The Young-HUNT Study: Young-HUNT1 (1995-97), Young-HUNT3 (2006-08), and Young-HUNT4 (2017-19). Each wave of the Young-HUNT data is conducted at a 10-year interval and comprises three independent adolescent population samples. To avoid repeated analyses of the same participants, the Young-HUNT2 survey, which is a 4-year follow-up of Young-HUNT1, was not included in this study. Almost all adolescents in Norway attend primary high schools (age 13–16 years) and upper secondary high schools (16–19 years). The Young-HUNT study includes students from 66 schools. Participants were invited using an invitation letter including thorough information about the study and use of data. The schools were visited by specially trained nurses for interviews and measurements. Students absent on the day of the questionnaire were encouraged to complete this when the nurses visited the schools. Adolescents not in school according to the records of the county school authorities were invited to the study by post. The flow chart for the number of participants in this study is shown in Fig. 1. The samples within each survey ranged from 8066 to 8980 participants.

### Variables

#### Loneliness

Loneliness was measured by using a single question in all three Young-HUNT waves asking, *‘Are you lonely?’* rated on a five-point scale *(1) Very rarely or never, (2) Rarely, (3) Sometimes, (4) Often, and (5) Very often.* The loneliness variable was dichotomized into the category Rarely lonely (‘Very rarely or never’ + ‘Rarely’ + ‘Sometimes lonely’) and the category Very lonely (‘Often’ + ‘Very often’). We measured loneliness by including those adolescents who reported being lonely often and very often. The ‘Very lonely’ category was presumed to capture the most severe and high-risk cases of loneliness, and the ‘Rarely lonely’ category captured less severe and temporary form or a complete absence of loneliness.

#### Mental distress

Mental distress was measured using the 5-item Hopkins Symptom Checklist (HSCL-5). Hopkins Symptom Checklist-25 (HSCL-25) is an extensively used self-report measure of anxiety and depression symptoms. Compared with the HSCL-25, the short-form model fit is good with acceptable validity [46]. The adolescents were asked if they had experienced each of the following during the last 14 days: *‘Been constantly afraid and anxious’, ‘Felt tense or uneasy’, ‘Felt hopelessness about the future’, ‘Felt dejected or sad’, ‘Worried too much about various things’.* Each item was answered on a four-point scale: *(1) Not at all*, *(2) A little*, *(3) Quite a bit, and (4) Very much* with the cut-off score ≥ 2. Cronbach’s alpha values for the HSCL-5 instrument were calculated in this study to be 0.789, 0.829, and 0.873 for wave 1, 2, and 3, respectively. HSCL-5 is referred to as HSCL from here on.

#### Self-rated health

Self-rated health was assessed by a single direct question, ‘How is your health at the moment?’ with four possible responses: *(1) Poor, (2) Not very good, (3) Good, and (4) Very good* and was categorized as Poor self-rated health (‘poor’ + ‘not very good’) and Good self-rated health (‘good’ + ‘very good’). The dichotomization of self-rated health has been done in previous studies among adolescents [47–49].

#### Sociodemographic factors

Sociodemographic characteristics of gender and age, socioeconomic status (SES), number of close friends, and parent’s civil status were used as covariates and adjusted for in the logistic regression model for each Young-HUNT survey. The selection of gender and age as covariates was based on the literature described above. According to previous studies, high-quality friendships are a critical resource against loneliness, and not having any close friends in adolescence was related to comparably high loneliness and depression [24, 29, 50]. Moreover, loneliness in children and adolescents is influenced by how well accepted they are by peers (social loneliness), whether they have friends, and the durability and quality of their best friendships (emotional loneliness) [51, 52]. A decrease in SES was associated with an increasing prevalence of moderate to high symptom load of psychological distress and loneliness [23]. Children coming from broken families with divorced or separated parents live in adaptive and flexible families and are forced by the circumstances of the divorce to adjust. Such circumstances and instability are likely to increase the child’s loneliness [25]. These variables were dichotomized and adjusted for in the multiple logistic regression model. Age groups were divided in two: 13–15.0 years and 15.1–19 years. Age groups are displayed as 13–15 years and 16–19 years in figures. SES was measured by adolescents’ future education plans in Young-HUNT wave 1. The variable included 5 categories: *(1) None*, *(2) College or university less than 4 years*, *(3) College or university for 4 years or more*, *(4) Vocational school or training and (5) Don’t know.* College or university education of fewer than 4 years and for 4 years or more was ‘high education’, which was a proxy for high SES. Education is frequently used as an indicator of SES in epidemiological studies [53, 54]. Self-report measures with the question ‘How well off do you think your family is compared to most others?’ were used to assess SES in Young-HUNT wave 3 and 4 with the categories *(1) About the same as most others, (2) Better financial situation, (3) Worse financial situation.* The first two categories were classified as ‘moderate to high SES,’ and the last category was ‘low SES.’ The number of close friends was a self-report measure across all three Young-HUNT waves. Adolescents who reported having one or several friends were considered in the category of those having close friendships. Parents’ civil status was assessed by adolescent self-report and was divided into 3 categories: *(1) Not divorced*, *(2) Separated but got back together, and (3) Divorced permanently*. Category 3 was considered as parents with a divorce.

### Statistical analysis

We used descriptive statistics to characterize the sample’s sociodemographic profile, loneliness levels, mental distress, and self-rated health individually for each Young-HUNT wave. The group of adolescents in the category “Very lonely” was compared between the three Young-HUNT waves by the following variables: (1) Gender (2) Age (3) Mental distress and (4) Self-rated health. Pearson’s chi-square test was used to investigate the differences between frequency distributions. To investigate time trends of loneliness, we tested the Linear-by-Linear association across survey years. Associations between mental distress, self-rated health, and the outcome of loneliness were examined using multiple logistic regression. Odds ratios (OR), corresponding 95% confidence intervals (95% CI), and p-values were calculated for all associations. The results were considered statistically significant when we had enough evidence to reject the null hypothesis, i.e., with a p-value < 0.05. The analyses were conducted separately for each Young-HUNT wave with mental distress and self-rated health as the main exposure variables. Fully adjusted models were examined, controlling for the potentially confounding sociodemographic factors. Mental distress and self-rated health were mutually adjusted in the models. All the variables were tested for multicollinearity by checking for correlations between the covariates. The group’ Very lonely’ was compared to the reference group ‘Rarely lonely’ in the analysis because a prolonged feeling of loneliness is associated with severe health problems [55]. The dichotomization has been applied in earlier adolescent studies [28, 56, 57]. For mental distress and self-rated, ‘HSCL score < 2’ and ‘Good self-rated health’ were used as reference categories, respectively. We tested for effect modification by gender and age for both exposures (i.e., mental distress and self-rated health), by including interaction terms in the adjusted models. All analyses were done using SPSS version 28.

**Table 1:** Characteristics of the sample population by survey year

SURVEY YEAR	Young-HUNT1 1995–1997		Young-HUNT3 2006–2008		Young-HUNT4 2017–2019	
	N	%	N	%	N	%
	8980	100	8199	100	8066	100
**GENDER**						
Girls	4464	49.7	4128	50.3	4108	50.9
Boys	4516	50.3	4071	49.7	3958	49.1
**AGE**						
13–15 years	3002	33.4	3083	37.6	2676	33.2
16–19 years	5978	66.6	5116	62.4	5390	66.8
**LONELINESS**						
Rarely lonely	8305	92.5	6908	84.3	6925	85.8
Very lonely	533	5.9	735	9.0	819	10.2
Missing	142	1.6	556	6.8	322	4
Total	8980	100	8199	100	8066	100
MENTAL DISTRESS (Hopkins Symptom Checklist – 5 (HSCL-5)) *						
Low (HSCL < 2)	7436	82.8	6465	78.9	5431	67.3
High (HSCL ≥ 2)	1404	15.3	1562	19.0	2423	29.8
*Girls*	945	21.4	1121	27.6	1790	44.5
*Boys*	459	10.4	441	11.1	633	16.5
*13–15 years*	322	10.9	428	14.2	561	21.6
*16–19 years*	1082	18.4	1134	22.6	1862	35.4
Missing	140	1.6	172	2.1	212	2.6
**SELF-RATED HEALTH**						
Poor self-rated health	968	10.8	874	10.7	1118	13.9
*Girls*	530	12.1	499	12.3	671	16.5
*Boys*	438	9.9	375	9.3	447	11.4
*13–15 years*	274	9.3	233	7.7	251	9.5
*16–19 years*	694	11.8	641	12.7	867	16.2
Good self-rated health	7859	87.5	7212	88	6868	85.1
Missing	153	1.7	113	1.4	80	1
**SOCIOECONOMIC STATUS**						
Low	6724	74.9	708	8.6	629	7.8
Moderate to high	1936	21.6	6928	84.5	7318	90.7
Missing	320	3.5	563	6.9	119	1.5
**CLOSE FRIENDS**						
No friends	143	1.6	113	1.4	100	1.2
One or more friends	8647	96.3	7628	93	7929	98.3
Missing	190	2.1	458	5.6	37	0.5
**PARENT’S CIVIL STATUS**						
Not divorced	6956	77.5	5396	65.8	5306	65.8
Divorced	1741	19.4	2234	27.2	2663	33
Missing	283	3.1	569	7	97	1.2

*Hopkins Symptom Checklist – 5 (HSCL-5) cut off score ≥ 2

## Results

### Study population characteristics

Table 1 shows the study participants’ characteristics within the three waves: Young-HUNT1, Young-HUNT3, and Young-HUNT4. The sample sizes were almost similar between boys and girls, and more adolescents from the 16–19-year age group participated in each survey year than those from the 13–15-year age group. The prevalence of adolescents reporting to be ‘Very lonely’ increased from 5.9% in 1995-97 to 10.2% in 2017-19 (P-value for trend < 0.05), with the highest increase between 1995-97 and 2006-08. The percentage of adolescents reporting mental distress nearly doubled between 1995-97 and 2017–19, from 15.3 to 29.8%, with the highest increase occurring between 2006-08 and 2017–19. Among the boys and girls participating in each survey, the prevalence of mental distress was 10.4% for boys and 21.4% for girls in the 1990s. In the latest survey (2017–2019), the prevalence changed to 16.5% for boys and 44.5% for girls. The prevalence of mental distress increased from 11 − 21.6% in 13-15-year-olds and from 18.4 to 35.4% in 16–19-year-olds in the period between 1995-97 to 2017-19. The prevalence of poor self-rated health remained stable at 10.8% and 10.7% in 1995–1997 and 2006–2008, respectively, while it increased to 13.9% in 2017-19. The prevalence of poor self-rated health increased from 12% in the 1990s to 16.5% in 2017-19 among girls and 10% in the 1990s to 11.4% in 2017-19 among boys. Among the 13–15-year-olds, the prevalence of poor self-rated health increased from 9.3% in the 1990s to 9.5% in 2017-19, while for the 16–19-year-olds, the increase changed from 11.8% in the 1990s to 16.2% in 2017-19.

### Prevalence trends of the ‘very lonely’ group by gender, age, mental distress, and self-rated health

**Fig. 2:**
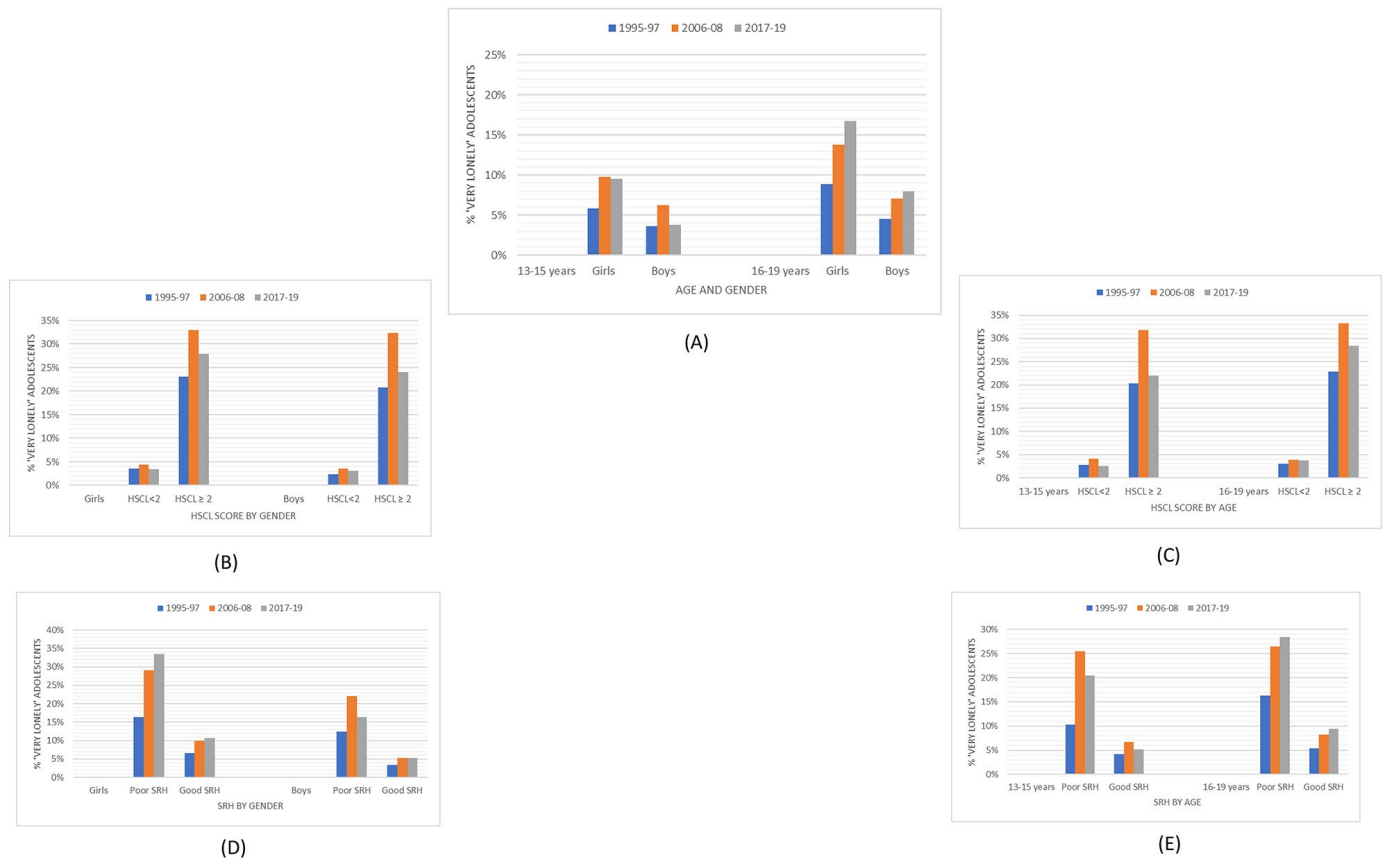
Prevalence (%) trends of Very lonely adolescents by **(A)** age and gender; **(B)** mental distress (HSCL < 2 and HSCL ≥ 2) and gender; **(C)** mental distress (HSCL < 2 and HSCL ≥ 2) and age; **(D)** self-rated health (SRH) and gender; **(E)** SRH and age from 1995–2017

When looking at the results for the `very lonely` group of adolescents, there was an increase in loneliness over time in girls from 7.8% in 1995-97 to 14.3% in 2017-19. Girls showed higher levels of loneliness than boys in all waves (p < 0.001). Among the 16–19-year-olds, loneliness prevalence increased from 6.7% in 1995-97 to 12.5% in 2017-19, (Table 2). When the loneliness prevalence trends were plotted by age and gender collectively, a higher prevalence was observed among girls than boys in both age groups. There was a gradual increase in the prevalence of loneliness among girls and boys of 16–19 years from 1995-97 to 2017-19, but the highest prevalence was seen in girls of 16–19 years overall. However, among girls and boys of 13–15 years, the highest prevalence was observed in 2006-08 (Table 2; Fig. 2A). A higher prevalence of loneliness was observed in adolescents with mental distress (HSCL ≥ 2) than among those without mental distress (HSCL < 2) across all survey years. An interesting finding was that the prevalence of loneliness, i.e., 32.8%, was the highest among those with HSCL ≥ 2 during 2006-08, followed by a decrease to 26.9% in 2017-19 (Table 2).

**Table 2:** Prevalence % and 95% CI of the “Very lonely” category of adolescents by gender, age, mental distress, and self-rated health

SURVEY YEAR	Young-HUNT1 1995–1997	Young-HUNT3 2006–2008	Young-HUNT4 2017–2019	P-values	
	Prevalence (%) 95% CI	Prevalence (%) 95% CI	Prevalence (%) 95% CI	P value for trend	Pearson’s Chi square test
**Gender**					
GIRLS	7.8 (7.0–8.6) (N = 4414)	12.3 (11.3–13.3) (N = 3932)	14.3 (13.3–15.3) (N = 3974)	< 0.001	< 0.001
BOYS	4.2 (3.6–4.8) (N = 4424)	6.8 (6–7.6) (N = 3711)	6.6 (5.8–7.4) (N = 3770)	0.039	
**Age**					
13–15 years	4.7 (3.9–5.5) (N = 2938)	8.1 (7.1–9.1) (N = 2796)	6.7 (5.7–7.7) (N = 2548)	0.008	< 0.001
16–19 years	6.7 (6.1–7.3) (N = 5900)	10.5 (9.6–11.4) (N = 4847)	12.5 (11.6–13.4) (N = 5196)	< 0.001	
**Gender/Age**	n (%)	n (%)	n (%)		
13–15 years					
Girls	85 (5.8) (N = 1474)	142 (9.8) (N = 1451)	123 (9.5) (N = 1301)	< 0.001	< 0.001
Boys	53 (3.6) (N = 1464)	84 (6.2) (N = 1345)	47 (3.8) (N = 1247)	0.722	
16–19 years					
Girls	261 (8.9) (N = 2940)	342 (13.8) (N = 2481)	447 (16.7) (N = 2673)	< 0.001	< 0.001
Boys	134 (4.5) (N = 2960)	167 (7.1) (N = 2366)	202 (8) (N = 2523)	< 0.001	
**Mental distress**					
Low (HSCL < 2)	2.9 (2.5–3.3) (N = 7404)	4.0 (3.5–4.5) (N = 6107)	3.2 (2.7–3.7) (N = 5330)	< 0.001	< 0.001
High (HSCL ≥ 2)	22.3 (20.1–24.5) (N = 1398)	32.8 (30.4–35.2) (N = 1493)	26.9 (25.1–28.7) (N = 2381)	0.003	
**Self-rated health**					
Poor self-rated health	14.6 (12.4–16.8) (N = 951)	26.3 (23.3–29.3) (N = 807)	26.6 (23.9–29.3) (N = 1068)	0.001	< 0.001
Good self-rated health	5.0 (4.5–5.5) (N = 7791)	7.6 (7–8.2) (N = 6751)	7.9 (7.3–8.5) (N = 6632)	0.503	

When stratified by mental distress, the prevalence of loneliness was marginally higher among girls and 16–19-year-olds compared to boys and 13–15-year-olds, respectively. Among both genders and age groups, loneliness levels peaked in 2006-08 (Fig. 2B, C). Adolescents who reported being very lonely increased with deteriorating self-rated health from 14.6% in 1995-97 to 26.6% in 2017-19, with the highest increase between 1995 and 97 and 2006-08 (Table 2). As seen in Fig. 2D, girls with poor self-rated health showed a gradual increase in loneliness prevalence over a 24-year period. Similar trends were observed in adolescents aged 16–19 years with poor self-rated health. Boys and adolescents of 13–15 years with poor self-rated health showed a peak in loneliness prevalence in the survey year 2006-08 (Fig. 2E). Although lesser than adolescents with poor self-rated health, those with good self-rated health also showed a gradual increase in loneliness prevalence over time. There were differences in loneliness prevalence between boys and girls, age groups, mentally distressed or not, and poor and good self-rated health with statistically significant trends (p < 0.005). The proportions of loneliness prevalence within the subgroups of mental distress and self-rated health are presented in tables in the Supplementary material.

### Associations of mental distress and self-rated health with loneliness

Table 3 shows the fully adjusted associations between mental distress, self-rated health, and the outcome of loneliness for each Young-HUNT survey. There was an increased risk of loneliness in those with mental distress (HSCL ≥ 2) in all three waves of the Young-HUNT survey, i.e., OR 7.57 (95% CI: 6.18–9.28); OR 9.15 (95% CI: 7.6–11.02) and OR 8.27 (95% CI: 6.8–10.05) for wave 1 (Young-HUNT1), wave 2 (Young-HUNT3) and wave 3 (Young-HUNT4) respectively. Higher risk of loneliness was also observed in adolescents with poor self-rated health compared to those with good self-rated health (OR 1.91 (95% CI: 1.51–2.42); OR 2.06 (95% CI: 1.67–2.55) and OR 2.0 (95% CI: 1.67–2.41) for wave 1 (Young-HUNT1), wave 2 (Young-HUNT3) and wave 3 (Young-HUNT4) respectively. No strong correlations were observed among the variables included in the models, and we found no evidence of effect modification of gender or age group (p > 0.05). The unadjusted odds ratios and 95% CI are presented in the Supplementary material.

**Table 3:** Logistic regression analyses showing the associations of mental distress and poor self-rated health with loneliness in the fully adjusted model

SURVEY YEAR	1995-97 (Young-HUNT1)		2006-08 (Young-HUNT3)		2017-19 (Young-HUNT4)	
	OR (95% CI)	P-value	OR (95% CI)	P-value	OR (95% CI)	P-value
Mental distress (HSCL < 2 vs. HSCL ≥ 2)	7.57 (6.18–9.28)	< 0.001	9.15 (7.6–11.02)	< 0.001	8.27 (6.8–10.05)	< 0.001
Self-rated health (poor vs. good)	1.91 (1.51–2.42)	< 0.001	2.06 (1.67–2.55)	< 0.001	2.0 (1.67–2.41)	< 0.001

Note: OR: Odds ratio; CI: Confidence interval

The analyses were adjusted for gender, age, SES, number of close friends, and civil status of parents. The variables of mental distress and self-rated health were mutually adjusted. The group ‘Rarely lonely’ (‘Very rarely or never’ + ‘Rarely’ + ‘Sometimes lonely’) was used as the reference group

## Discussion

This study presents findings from one of the largest population-based studies in Norway, including more than 25,000 adolescents aged 13–19 years. The outcomes from this study are consistent with all three hypotheses we made. One of the interesting main findings shows that the prevalence of adolescents reporting to be very lonely almost doubled from 5.9% in 1995-97 to 10.2% in 2017-19. This is consistent with recent studies from the Nordic countries. For example, recent Norwegian national reports show that 10–11% of adolescents report feeling ‘very lonely’ [13]. Furthermore, Madsen et al. [14] and Lyrra et al. [20] reported an increase in adolescent loneliness in Denmark from 4.4% in 1991 to 7.2% in 2014 and in Finland from 11% in 2006 to 15% in 2018. The overall increase in loneliness prevalence over time is complex and is likely to be explained by different factors. One factor may be related to the persistent increases in adolescent mental health problems, which might mirror greater openness and self-reporting in more recent cohorts. This may have resulted in a shift in thresholds or a greater sensitivity to symptoms and problematic behaviours due to today’s societal norms, previously seen as conventional [58]. Secondly, recent decades, as compared to earlier ones, have shown considerable changes in children’s social and surrounding environments as they grow up, which contribute to the present trends in mental health problems. These include e.g., factors associated with changes in individual vulnerability, family life, pressure at school, and broader socioeconomic and cultural factors [15, 58]. Our findings align with a study by Twenge et al. [17], showing a global increase in loneliness among adolescents over the last decade and nearly twice as many adolescents in 2018 vs. 2012 with elevated levels of school-related loneliness. Similarly, research shows that self-reported loneliness has risen considerably among Nordic youth, particularly 15–16-year-olds, since 2000 [59, 60]. A suggested contributing factor to these trends is the broader access to smartphones and expanding internet use [17, 40, 59].

The prevalence of loneliness was higher among girls compared to boys across all surveys, with the highest prevalence among 16–19-year-old girls in the period 2017-19 (Young-HUNT4). There was also a rise in prevalence among boys over time, but this increase was smaller than for girls, and the highest level of loneliness among boys was observed in 2006-08 (Young-HUNT3, wave two). These results are coherent with previous findings showing that girls report higher levels of loneliness than boys do [28], and the most reported type of loneliness among girls is social loneliness [7, 61]. Boys seem to report higher levels of emotional loneliness during childhood [6, 62] and adolescence [7, 63]. Different personal and contextual factors can also explain these gender differences in loneliness patterns. Girls report higher levels of internalizing problems such as anxiety and depression—which correlate highly with feelings of loneliness [3, 64]. With growing age, girls might also be more susceptible to social comparison and social pressure than boys [65]. Moreover, girls are perhaps more predisposed to loneliness due to their tendency to report lower self-esteem and to critically evaluate themselves [19, 66]. The Norwegian national reports show that school pressure is reported to be higher in girls than boys [67]. Adolescent girls thus suffer an elevated stress level due to the build-up of worries about success in education and personal issues affecting their mental health [68]. Another explanation could be that girls and boys have different response patterns on questionnaires. Males report higher loneliness rates with indirect loneliness measuring scales [69], while females are more likely to disclose and speak more openly about being lonely when using direct measures and self-labelling [8, 70, 71]. Although lower than in girls, the results of this study also point to a steadily increasing prevalence pattern of loneliness among boys aged 16–19 years from 1995-97 to 2017-19. Boys generally have more externalizing problem behaviour [72] and may be more likely to shy away from their feelings of loneliness due to their fear of the possible stereotyping and stigma associated with loneliness [28]. Emotional loneliness seen in boys is a more guarded and hidden emotion than among girls. Moreover, behavioural tendencies to downplay emotions might predispose boys to emotional loneliness if they do not have someone to confide in [61]. Findings on the Norwegian adolescent population show that the reporting of loneliness increases from lower-secondary to upper-secondary school [13]. Previous studies have revealed that the transition to high school is associated with increased loneliness [73]. Some of the reasons could be related to social relationships due to peer status [74], bullying, and victimization [75]. These factors thus explain the increase in mid and late-adolescent loneliness. This study shows that among the age group 13–15 years, loneliness prevalence increased from 1995-97 to 2006-08 among girls and boys and remained stable until 2019 among girls. However, loneliness prevalence decreased from 2006-08 to 2017-19 among boys. This finding aligns with the study conducted by Van Roekel et al. [76], which state that the reasons for these gender difference patterns remain unclear and intriguing.

The current study found that the prevalence of loneliness among adolescents in the group with mental distress (HSCL ≥ 2) increased from wave one (Young-HUNT1) to wave two (Young-HUNT3) followed by a decrease in wave three (Young-HUNT4). Moreover, mental distress symptoms were strongly associated with adolescent loneliness in the adjusted analyses in all three waves with high OR of 7.57 (95% CI: 6.18–9.28); OR 9.15 (95% CI: 7.6–11.02) and OR 8.27 (95% CI: 6.8–10.05) for wave 1 (Young-HUNT1), wave 2 (Young-HUNT3) and wave 3 (Young-HUNT4) respectively. Similar findings showing the same strength of association between depression and mental health were seen with loneliness in previous studies [77, 78]. The effect sizes derived in these studies indicated that the relationship between depression and loneliness was in the range of a large effect size for studies for 33 hypotheses derived from 30 studies (r = 0.61 to 0.62) [77]. Similarly, the adjusted coefficient of full (R2) or partial determination (Rp2) was used to assess the goodness of fit, showing Rp2 = 10.55%, CI (7.59%, 14.2%) indicating a strong association between mental health and loneliness (N = 2240) [78]. These findings were also in line with Young’s cognitive theory, which suggests that individuals blame their faults for the weaknesses of their social relationships [79]. Moreover, depressed people focus more on the negative aspects of their relationships and, therefore, are more likely to feel lonely. Thus, adolescents with mental distress symptoms may have increased social withdrawal and vice versa [35]. Chronic and frequent symptoms of depression, such as anhedonia, low energy, and hopelessness, can burden interpersonal relationships [80]. This can lead to an abandonment of relationships and social isolation [35, 81, 82]. This, in turn, intensifies the feelings of loneliness, bringing about negative or distressing emotions that might accompany the perception that one’s social needs are not being adequately met. The bi-directionality of the relationship between depressive symptoms and loneliness may result in persistent symptoms of both.

We also found that adolescents with poor self-rated health had a higher prevalence of loneliness. The prevalence of loneliness in this group increased from wave one (Young-HUNT1) to wave two (Young-HUNT3) followed by a decrease in wave three (Young-HUNT4) among the 13–15-year-olds. Furthermore, those with poor self-rated health have almost twice the increased risk of experiencing loneliness. These findings are in line with previous studies showing that the association between loneliness and subjective health appears to be bidirectional. For example, loneliness may adversely impact health and well-being through a variety of behavioural and physiological pathways [42, 43], while poor or worsening health may hinder social interaction causing the onset or intensification of feelings of loneliness [44, 83]. For example, Hajek et al. found an effect size of (β = 0.04, p < 0.001) in the association between self-rated health and loneliness in a sample population of 101,909 older adults [44]. Furthermore, loneliness has been linked with reduced engagement in a healthy lifestyle (e.g., healthy diet and exercise) and lowered sleep quality [84, 85]. There is, however, a lack of such studies among adolescents, and future research is needed. Mental health is an essential element of self-rated health. In fact, self-rated mental health is found to be two times more important than self-rated physical health in predicting health [86]. The findings in this study concerning the deteriorating trends of mental health among adolescents are thus a possible explanation for the declining self-rated health and a consequent increase in loneliness. However, limited longitudinal work has examined temporal dynamics between loneliness and health among the adolescent population, and further research is needed to delimit the underlying mechanisms.

The results from this study point to a new finding that the prevalence of loneliness peaked in 2006-08 and gradually declined in 2017-19, especially among 13–15-year-old boys, adolescents with mental distress, and boys 13–15-year-olds with poor self-rated health. We will highlight a few possible theories explaining these trends for future studies. The 24-year period investigated in the present study has been recognized as a period with increasing social inequalities mirroring the increasing trends of mental health problems and associated loneliness among the young. Social inequalities play a significant role in children’s overall access to and use of material, cognitive, socio-emotional, and health resources, which impact their overall development and well-being from a life course perspective [87, 88]. This can be explained by the fact that there is a link between family SES, family structure and different family stressors and children’s socioemotional development, mental health and adjustment and participation in society [89–95]. Thus, the atypical increase of loneliness prevalence in 2006–2008 in this study could possibly be explained by the clear socioeconomic gradient over the last two decades related to these growing inequalities in Norway. However, this does not correspond with the decreasing level of loneliness from 2006/08 to 2017/19. A possible explanation for this observed nonlinear pattern and declining loneliness trends within these subgroups could be the increased use of social media over the years. In the observation period 1995–2017, adolescents have increasingly used digital devices and social media. Social media provides access to social networks and groups which give support and belongingness. This may help cope with physical and mental health problems [96]. However, conclusive statements on the impact of increased internet use on loneliness cannot be made, as ambiguous findings and cross-sectional studies dominate the research on this topic. We need, therefore, further reflection and research to understand the causalities behind the different trends of loneliness among adolescents.

### Strengths and limitations

A main strength of the study is its large, nationally representative samples, which allowed us to explore long term trends in loneliness among Norwegian adolescents on a substantial scale. The Young-HUNT Study is a population-based longitudinal study collecting 10-yearly data. This facilitates researchers to examine the population’s health changes and prevalence trends over time [97, 98]. The sampling of participants and methods for measuring loneliness, mental distress, and self-rated health have been conducted using the same methods and instruments in all three surveys included in the present study. The population is stable and relatively homogenous, with a low migration. As part of a national Nordic welfare state, the population recruited is part of a country with universal public health and welfare services and a school system where almost everyone attends the same public schools. Moreover, this is one of the few studies that examined loneliness over such a long study period, and representative population samples are compared using identical symptom screens over all three survey points. Another strength is the scope of explanatory variables chosen in the analyses. This contributes to identifying potential risk factors for loneliness, which could affect health promotion and prevention programs.

However, it is essential to address the limitations. Firstly, the measures used in this study were self-reported. This may subject the study to potential challenges concerning self-report bias, for example, social desirability, over- and under-reporting due to potential social stigma, and gender role bias. Secondly, non-participation may result in selection bias despite the high participation rate. The number of missing cases in wave 2 (Young-HUNT3) exceeded the other two waves. A few potential reasons could have been the placement of the question on loneliness in the questionnaire and the time given. Since the loneliness question is placed quite far into the Young-HUNT3 questionnaire and the forms were completed in just one school hour, it may be that some of the young people did not get time to answer. It is also possible that those students who were not present on the day of data collection had a high grade of loneliness and physical or mental distress. That means the analyses may have underestimated the prevalence of loneliness and its associations.

Moreover, adolescents not attending school due to any reason are poorly represented. Thirdly, the questions used for obtaining data on SES are inconsistent and subjective over the three surveys. The question on education plans in wave 1 (Young-HUNT1) was used as a proxy for SES. However, we performed the analyses with and without the SES variable as a confounder to be more specific on the associations of loneliness. The cross-sectional design is a limitation of this study, precluding it from making causal conclusions. The span of 24 years covered by the data, in combination with the limited number of measurement waves, might have influenced the results. Shorter time intervals between the measurement waves would have yielded more reliable results. Fourthly, the measurement of loneliness may result in information bias. Questions asking directly about loneliness may result in under-reporting of the prevalence of loneliness, given the social stigma associated with loneliness [98]. Loneliness is a complex experience, and its assessment may be difficult with one item. It should, therefore, be measured by an instrument that includes variations in intensity, circumstances, and time, as well as both direct and indirect questions. Therefore, the reported prevalence of loneliness would likely appear different if we had used a multi-item scale. The rationale behind using the loneliness question in this study was that the Young-HUNT study inherently has only one direct question over all waves to measure loneliness.

Nevertheless, the strength of this approach is that one similar question has been used consistently for all waves of the study, enabling authors to deduce conclusions over an extensive 24-year period. Another validity issue is the categorization and applied dichotomization of loneliness into the categories ‘very often’ and ‘often’ versus the categories ‘sometimes,’ ‘rarely’ and ‘very rarely or never.’ However, it is reassuring that this same dichotomization has been applied in earlier research among adolescents [14, 56, 57]. Nevertheless, despite the broad ranking and categorization of the loneliness variable in this study, the variable does not differentiate between the adolescents who are rarely lonely and those who are never lonely. Lastly, the variable ‘Close friends’ does not capture the quality and intensity of adolescents’ friendships. Despite these limitations, these results will have adequate implications for the public health of adolescents in Norway, given the extended study period and the sizeable homogenous sample.

### Implications and future Research

From a research perspective, it is vital to gain increased knowledge of the processes that cause changing levels of loneliness in the adolescent population. Developing interventions and health promotion efforts to prevent loneliness among youth is vital. Our findings show that feelings of loneliness are more severe in those who report poor self-rated health and mental health. A better understanding of the factors that increase the risk of loneliness in young people with mental distress would contribute to developing effective interventions. Early interventions in controlling feelings of loneliness are critical to prevent lonely adolescents from being blocked with feelings of loneliness as they grow older [99]. It is, however, more important to be one step ahead and focus on creating suitable upbringing environments for adolescents that promote belongingness, presumably preventing loneliness. School may be an ideal setting to target the entire population of adolescents in addressing their mental health and implementing universal actions. In combination with a focus on creating a positive psychosocial school environment, preventive measures may also improve prospects for adolescents’ well-being, future academic performance, and mental health. The burden of loneliness symptoms in this age group must be acknowledged as a rising public health problem, and the future holds the need to increase efforts focused on health promotion and prevention strategies. As there was a lack of ethnic diversity and the cultural dimensions of social connections in our sample, we also need studies conducted in different ethnic groups. Future studies can investigate longitudinal associations between loneliness and different health outcomes and potential protective factors moderating these associations.

## Conclusion

Our results show an overall trend of significantly increasing loneliness among adolescents over a 24-year period, particularly among 16–19-year-old girls. This increase mirrors and associates with the deteriorating mental and self-rated health trends over three decades in this population sample. Secondly, an initial increase in loneliness prevalence in the first two decades, followed by a steady decrease in the last decade, was observed. These results highlight the importance of developing health-promoting environments for children and adolescents, promoting connectedness, social support, integration, and belongingness in their daily lives.

### Electronic supplementary material

Below is the link to the electronic supplementary material.

Supplementary Material 1

## Data Availability

The HUNT databank provided the data used in this study, but access is restricted. Data can be accessed by approaching the corresponding author, Nayan D. Parlikar (nayan.d.parlikar@ntnu.no) and permission from HUNT, The Regional Ethical Committee, and the Norwegian Data Protection Authority. All the subjects of this study are stored in the HUNT data using a personal identification number as an I.D. number. The HUNT Research Centre stores and uses these data with authorization from the Norwegian Data Inspectorate without breaking participant privacy. The researcher will always receive an anonymous dataset after approval from the Regional Ethical Committee and HUNT Research Centre. For more information about HUNT data see https://www.ntnu.edu/hunt/data.

## Abbreviations

HUNT: Health Study of Trøndelag
SRH: Self-rated health
HSCL: 25-Hopkins Symptom Checklist-25
HSCL: 5-Hopkins Symptom Checklist-5
SES: Socioeconomic status
CI: Confidence interval
OR: Odds ratio
REK: Regional Committee for Ethics in Medical Research
