# Supplementary material for: Loneliness in the Norwegian adolescent population: prevalence trends and relations to mental and self-rated health
Source: BMC Psychiatry. 2023 Nov 30;23:895. doi: 10.1186/s12888-023-05404-5 (PMC10688064; doi:10.1186/s12888-023-05404-5)
Supplement: Supplementary file 1 — Supplementary Material 1 [file 12888_2023_5404_MOESM1_ESM.docx]

Supplementary Material

**Loneliness in the Norwegian adolescent population: prevalence trends and relations to mental and self-rated health**

Nayan Parlikar^1*^, Kirsti Kvaløy^2,3,4^, Linn Beate Strand^1^, Geir Arild Espnes^1^, Unni Karin Moksnes^1^

^1^Department of Public Health and Nursing, Faculty of Medicine and Health Sciences, Norwegian University of Science and Technology, Trondheim, Norway

^2^HUNT Research Centre, Department of Public Health and Nursing, Faculty of Medicine and Health Sciences, Norwegian University of Science and Technology, Levanger, Norway

^3^Levanger Hospital, Nord-Trøndelag Hospital Trust, Levanger, Norway

^4^Centre for Sami Health Research, Department of Community Medicine, UiT - The Arctic University of Norway

*** Correspondence: Nayan Parlikar**Corresponding Author
[nayan.d.parlikar@ntnu.no](mailto:nayan.d.parlikar@ntnu.no)

**Table A1:** Prevalence (%) and 95% CI of loneliness among adolescents by gender and mental health and age and mental health in The Young-HUNT Study

| Hopkins score/Gender | Young-HUNT1  1995-1997 | | Young-HUNT3  2006-2008 | | Young-HUNT4  2017-2019 | |
| --- | --- | --- | --- | --- | --- | --- |
| GENDER | | | | | | |
| Girls | *N* | *%* | *N* | *%* | *N* | *%* |
| Hopkins score < 2 |  |  |  |  |  |  |
|  | 3455 | 100 | 2836 | 100 | 2196 | 100 |
| Very lonely | 125 | 3.6 | 126 | 4.4 | 75 | 3.4 |
| Hopkins score ≥ 2 | *N* | *%* | *N* | *%* | *N* | *%* |
|  | 941 | 100 | 1080 | 100 | 1764 | 100 |
| Very lonely | 217 | 23.1 | 356 | 33 | 493 | 27.9 |
|  | | | | | | |
| Boys | *N* | *%* | *N* | *%* | *N* | *%* |
| Hopkins score < 2 |  |  |  |  |  |  |
|  | 3949 | 100 | 3271 | 100 | 3134 | 100 |
| Very lonely | 91 | 2.3 | 116 | 3.5 | 97 | 3.1 |
|  | *N* | *%* | *N* | *%* | *N* | *%* |
| Hopkins score ≥ 2 |  |  |  |  |  |  |
|  | 457 | 100 | 413 | 100 | 617 | 100 |
| Very lonely | 95 | 20.8 | 134 | 32.4 | 148 | 24 |
| AGE | | | | | | |
| Hopkins score/age | **Young-HUNT1**  **1995-1997** | | **Young-HUNT3**  **2006-2008** | | **Young-HUNT4**  **2017-2019** | |
| 13-15 years | *N* | *%* | *N* | *%* | *N* | *%* |
| Hopkins score < 2 |  |  |  |  |  |  |
|  | 2604 | 100 | 2378 | 100 | 1993 | 100 |
| Very lonely | 72 | 2.8 | 98 | 4.1 | 50 | 2.5 |
|  | *N* | *%* | *N* | *%* | *N* | *%* |
| Hopkins score ≥ 2 |  |  |  |  |  |  |
|  | 320 | 100 | 400 | 100 | 541 | 100 |
| Very lonely | 65 | 20.3 | 127 | 31.8 | 119 | 22 |
|  | | | | | | |
| 16-19 years | *N* | *%* | *N* | *%* | *N* | *%* |
| Hopkins score < 2 |  |  |  |  |  |  |
|  | 4800 | 100 | 3729 | 100 | 3337 | 100 |
| Very lonely | 144 | 3 | 144 | 3.9 | 122 | 3.7 |
|  | *N* | *%* | *N* | *%* | *N* | *%* |
| Hopkins score ≥ 2 |  |  |  |  |  |  |
|  | 1078 | 100 | 1093 | 100 | 1840 | 100 |
| Very lonely | 247 | 22.9 | 363 | 33.2 | 522 | 28.4 |

**Table A2:** Prevalence (%) and 95% CI for loneliness among adolescents by gender and self-rated health and age and self-rated health in The Young-HUNT Study

| Self-Rated Health/Gender | Young-HUNT1  1995-1997 | | Young-HUNT3  2006-2008 | | Young-HUNT4  2017-2019 | |
| --- | --- | --- | --- | --- | --- | --- |
| GENDER | | | | | | |
| Girls | N | % | N | % | N | % |
| Poor self-rated health |  |  |  |  |  |  |
|  | 525 | 100 | 477 | 100 | 644 | 100 |
| Very lonely | 86 | 16.4 | 139 | 29.1 | 215 | 33.4 |
| Good self-rated health | N | % | N | % | N | % |
|  | 3836 | 100 | 3398 | 100 | 3308 | 100 |
| Very lonely | 255 | 6.6 | 337 | 9.9 | 350 | 10.6 |
|  | | | | | | |
| Boys | N | % | N | % | N | % |
| Poor self-rated health |  |  |  |  |  |  |
|  | 426 | 100 | 330 | 100 | 424 | 100 |
| Very lonely | 53 | 12.4 | 73 | 22.1 | 69 | 16.3 |
| Good self-rated health | N | % | N | % | N | % |
|  | 3955 | 100 | 3353 | 100 | 3324 | 100 |
| Very lonely | 131 | 3.3 | 177 | 5.3 | 177 | 5.3 |
| AGE | | | | | | |
| Self-rated health/age | **Young-HUNT1**  **1995-1997** | | **Young-HUNT3**  **2006-2008** | | **Young-HUNT4**  **2017-2019** | |
| 13-15 years | N | % | N | % | N | % |
| Poor self-rated health |  |  |  |  |  |  |
|  | 262 | 100 | 208 | 100 | 240 | 100 |
| Very lonely | 27 | 10.3 | 53 | 25.5 | 49 | 20.4 |
|  | N | % | N | % | N | % |
| Good self-rated health |  |  |  |  |  |  |
|  | 2624 | 100 | 2552 | 100 | 2287 | 100 |
| Very lonely | 110 | 4.2 | 171 | 6.7 | 119 | 5.2 |
|  | | | | | | |
| 16-19 years | N | % | N | % | N | % |
| Poor self-rated health |  |  |  |  |  |  |
|  | 689 | 100 | 599 | 100 | 828 | 100 |
| Very lonely | 112 | 16.3 | 159 | 26.5 | 235 | 28.4 |
|  | N | % | N | % | N | % |
| Good self-rated health |  |  |  |  |  |  |
|  | 5149 | 100 | 4199 | 100 | 4345 | 100 |
| Very lonely | 276 | 5.4 | 343 | 8.2 | 408 | 9.4 |

**Table A3:** Unadjusted associations of gender, age, mental distress, and self-rated health with loneliness among adolescents

|  | Unadjusted OR (95% CI) | P-value |
| --- | --- | --- |
| *1995-97 (Young-HUNT1)* | | |
| Gender | | |
| Girls vs boys | 1.93 (1.61 – 2.31) | <0.001 |
| Age | | |
| 13–15-year vs 16–19-year | 1.46 (1.19 – 1.78) | <0.001 |
| Socioeconomic status | | |
| Plans for low education vs. higher education | 0.69 (0.56 – 0.84) | <0.001 |
| Close friends | | |
| No friends vs one or more friends | 8.71 (6.08 – 12.48) | <0.001 |
| Parent’s civil status | | |
| Divorced vs not divorced | 1.49 (1.21 – 1.82) | <0.001 |
| Mental distress | | |
| HSCL ≥ 2 vs. HSCL <2 | 9.56 (7.95 – 11.5) | <0.001 |
| Self-rated health | | |
| Poor vs. good self-rated health | 3.28 (2.67 – 4.03) | <0.001 |
| *2006-08 (Young-HUNT3)* | | |
| Gender | | |
| Girls vs boys | 1.94 (1.65 – 2.27) | <0.001 |
| Age | | |
| 13–15-year vs 16–19-year | 1.33 (1.13 – 1.57) | <0.001 |
| Socioeconomic status | | |
| Low vs moderate to high | 4.29 (3.55 – 5.18) | <0.001 |
| Close friends | | |
| No friends vs one or more friends | 8.51 (5.72 – 12.66) | <0.001 |
| Parent’s civil status | | |
| Divorced vs not divorced | 2.01 (1.72 – 2.35) | <0.001 |
| Mental distress | | |
| HSCL ≥ 2 vs. HSCL <2 | 11.84 (10.01 – 14.01) | <0.001 |
| Self-rated health | | |
| Poor vs. good self-rated health | 4.32 (3.61 – 5.18) | <0.001 |
| *2017-19 (Young-HUNT4)* | | |
| Gender | | |
| Girls vs boys | 2.37 (2.02 - 2.77) | <0.001 |
| Age | | |
| 13–15-year vs 16–19-year | 2 (1.67 – 2.38) | <0.001 |
| Socioeconomic status | | |
| Low vs moderate to high | 3.45 (2.82 – 4.21) | <0.001 |
| Close friends | | |
| No friends vs one or more friends | 6.62 (4.34 – 10.1) | <0.001 |
| Parent’s civil status | | |
| Divorced vs not divorced | 1.67 (1.44 – 1.94) | <0.001 |
| Mental distress | | |
| HSCL ≥ 2 vs. HSCL <2 | 11.05 (9.26 – 13.19) | <0.001 |
| Self-rated health | | |
| Poor vs. good self-rated health | 4.2 (3.57 – 4.94) | <0.001 |
